# Elevated serum miR-142-5p correlates with ischemic lesions and both NSE and S100β in ischemic stroke patients

**DOI:** 10.1515/med-2024-1015

**Published:** 2024-12-09

**Authors:** WeiWei Xu, YongXia Cheng, Ning An, MeiLing Jiang

**Affiliations:** Department of Neurology 3, Hongqi Hospital Affiliated to Mudanjiang Medical College, Mudanjiang, Heilongjiang, 157011, China; Mudanjiang Medical College, Mudanjiang, Heilongjiang, 157011, China; Department of Neurology 2, Hongqi Hospital Affiliated to Mudanjiang Medical College, Mudanjiang, Heilongjiang, 157011, China; Department of Neurology 2, Hongqi Hospital Affiliated to Mudanjiang Medical College, No. 5, Tongxiang Road, Aimin District, Mudanjiang, Heilongjiang, 157011, China

**Keywords:** miR-142-5p, ischemic stroke, NSE, S100β

## Abstract

**Background:**

This study aims to evaluate the correlation between miRNAs and known nerve injury markers neuron-specific enolase (NSE) and S100β in ischemic stroke (IS) patients, exploring its efficacy.

**Methods:**

We retrospectively analyzed 86 IS patients and 32 healthy controls. Clinical and neurological examinations were performed in the admitted patients and the severity of neurological deficits was assessed by National Institutes of Health Stroke Scale (NIHSS). Plasma extraction and serum isolation were performed on all subjects before and 2 weeks after admission. miR-142-5p in serum, and NSE and S100β contents were measured by RT-qPCR and ELISA.

**Results:**

Ischemic lesions were more severe in IS patients, and NSE and S100β were abnormally elevated. miR-142-5p in the serum of IS patients was 2.85 times higher. After 2 weeks of treatment, serum miR-142-5, NSE, and S100β decreased. Patients’ serum levels of miR-142-5p were 57.5% lower. Serum miR-142-5, NSE, and S100β were lower in patients with disease improvement than in patients with poor recovery. Additionally, miR-142-5 was positively correlated with NSE (*P* < 0.0001) and S100β (*P* = 0.0147), and also with the NIHSS score (*P* = 0.0004).

**Conclusions:**

miR-142-5p, NSE, and S100β in peripheral blood (PB) of IS patients are elevated, and miR-142-5p is positively correlated with NSE and S100β.

## 1 Introduction

The number of deaths from stroke has increased over the last decade [1]. Although the mortality rate caused by stroke has decreased significantly in recent years, cerebrovascular diseases are still a global health burden [2]. Ischemic strokes (IS) are the most common type of stroke. It is caused by blood clots that block arteries and reduce blood pressure, leading to hypoperfusion or systemic hypoxia, which can cause irreversible damage to the brain. Current ischemic stroke treatment mainly includes intravenous thrombolysis, vascular intervention, antiplatelet, lipid modulation, free radical scavenging, neuroprotection, etc. [3,4], but current treatment methods and approaches are still inadequate and limited.

NSE is an acidic protease located in neurons and involved in the regulation of neuronal glycolysis [5]. NSE is a specific marker of neuronal injury. Increased blood NSE concentration is positively correlated with the severity of brain injury caused by cerebral ischemia [6]. In addition, S100β is a small molecular weight acidic calcium-binding protein with neurotrophic effects. S100β is a potential candidate biomarker for brain tissue injury and is released into the blood after IS in neurons, myelin, and glial cells [7]. It has been shown that patients experience damage to the peripheral ischemic hemianopsia and central necrotic zone due to lack of blood supply, resulting in neuronal cell damage. At this time, local brain tissues such as neuronal cells, glial cells, and blood vessels also become necrotic, leading to the release of NSE and S100β into the bloodstream from the damaged brain tissues [8,9]. As a result, patients had high serum NSE and S100β levels. NSE and S100β in peripheral blood (PB) can provide valuable and timely diagnostic information for stroke, which is necessary for timely management and decision-making, and is conducive to clinical diagnosis and treatment guidance for stroke patients.

Clinical diagnosis and treatment of IS complicated, and it is mainly diagnosed by relevant hematological markers combined with cranial imaging. However, there has been a lack of serological markers for screening, diagnosis, and prognostic evaluation of IS, resulting in some patients not being able to be prevented, intervened, and treated in a timely manner, resulting in irreversible neurological damage and even life-threatening. These small non-coding RNAs, miRNAs can target the 3′-untranslated region of mRNA and negatively regulate gene expression during the post-transcriptional process [10,11]. miRNAs are also involved in the progression of stroke and has become new therapeutic targets [12,13]. In this study, miR-126, miR-137, miR-142-5p, and miR-493 were included for study. miRNAs are dysregulated in IS and affect pathogenesis by modulating MAPK pathway regulation. Specifically, miR-137, miR-493, and miR-126 act as regulators of inflammation, apoptosis, angiogenesis, and neurogenesis mechanisms to limit permanent neuronal damage by regulating several target genes involved in IS [14]. In addition, it has been shown that miR-142 is involved in CNS-related diseases, and circHECTD1 is involved in the pathology of stroke by inhibiting the activity of miR-142 [15]. miR-142-3p is significantly downregulated in IS. Further, miR-142-3p expression is downregulated in various stroke subtypes [16]. miR-142-5p is localized on human chromosome 17 and is able to bind to the 3′ non-coding region of downstream target gene mRNAs and regulate gene expression at the post-transcriptional level. miR-142-5p is mostly used to study the mechanism of action in malignant tumors such as pancreatic cancer and lung cancer, and its down-regulation can lead to overexpression of downstream oncogenes. miR-142-5p has been proven to be associated with inflammation, apoptosis, and oxidative stress after lung ischemia/reperfusion injury and is involved in the regulation of cerebral ischemia–reperfusion injury and cognitive impairment [17]. Meanwhile, miR-142-5p is involved in the regulation of neuronal damage [18]. However, the relationship between miR-142-5p levels in IS patients and the neurological damage biomarkers NSE and S100β remains unclear.

In this study, we collected and analyzed biomarkers related to neurological injury and miRNAs related to inflammation in IS patients before admission and after treatment, and screened miR-142-5p, NSE, and S100β in the serum of IS patients for analysis. We further explored the correlation between miR-142-5p expression levels and National Institutes of Health Stroke Scale (NIHSS) scores, NSE, and S100β.

## 2 Materials and methods

### 2.1 Patients

From May 2016 to December 2020, a total of 86 IS patients, all with first-time IS, were enrolled in Hongqi Hospital Affiliated to Mudanjiang Medical College and voluntarily agreed to participate. IS diagnosis was established based on the patient’s chief complaint, history data, neurological findings, computed tomography and magnetic resonance imaging, and in accordance with the 2018 American Stroke Association/American Heart Association Guidelines for the Early Management of Patients with Acute Ischemic Stroke [19], and the severity of neurological deficits was assessed using NIHSS. Imaging revealed ≥50% stenosis of the lumen of large intracranial and extracranial arteries corresponding to neurological deficits in IS, and the presence of risk factors or evidence of atherosclerosis. In addition, brain diffusion-weighted imaging (DWI) information was acquired before and after treatment using a magnetic resonance scanner utilizing the DWI function of the system, with image layers of 5 mm thickness and a layer spacing of 1 mm. Lesion volume and the longest diameter of the lesion were measured on the DWI images. The ischemic lesion volume was the sum of the areas of high signal foci at all levels × (layer thickness + layer spacing). Exclusion criteria included: (1) cerebrospinal fluid infection, (2) autoimmune disease, (3) chronic kidney disease, and (4) head trauma or tumor. To eliminate potential errors, 32 healthy controls matching the age and gender of IS patients were recruited. Healthy people who had their medical checkups at the hospital during the same period and whose age and gender matched the cases were selected. This study follows the rules of the STROBE statement.

### 2.2 PB collection

Before admission and 2 weeks after admission, all patients and controls were subjected to PB extraction, and PB was collected in tubes anticoagulant with EDTA. Serum was prepared by centrifugation at 3,000 rpm for 10 min, collected into the centrifuge tubes, and stored at −80°C.

### 2.3 RT-qPCR

Total RNA was extracted from 200 μL serum samples using a miRNA purification kit (CW0627; CW Biotechnology, Beijing, China) and its concentration and quality were assessed using NanoDrop2000. Reverse transcription was performed using Superscript II (Invitrogen, USA), followed by PCR using SYBR Green Universal Master Mix reagent (Roche, USA). U6 expression was calculated by 2^−ΔΔCt^ method as the internal reference of miR-142-5p. U6: F: 5′-CTCGCTTCGGCAGCACA-3′, R: 5′- AACGCTTCACGAATTTGCGT-3′; miR-142-5p: F: 5′-AACTCCAGCTGGTCCTTAG-3′, R: 5′-TCTTGAACCCTCATCCTGT-3′. Amplification curves were generated by plotting the fluorescence signal and the number of cycles.

### 2.4 ELISA experiment

NSE and S100β contents were evaluated using ELISA kits (Beyotime, China) and determined by reading the optical density at 450 nm by a microplate reader (Thermo, USA). A standard curve of sample concentration was plotted and a linear regression equation was calculated from the OD values. The OD values were substituted and calculated for NSE and S100β.

### 2.5 Data analysis

Statistical analysis was conducted using GraphPad Prism7. Quantitative information that conforms to a normal distribution was shown in the form of mean ± standard deviation, with comparison among groups conducted via Student’s *t*-test. Qualitative information was expressed as number of cases or percentage using chi-square test. One-way ANOVA was used for three and more groups. Data that did not fit a normal distribution were described using the median and interquartile spacing [*M*(Q25, Q75)], and comparisons were made using the nonparametric rank sum test. The Pearson correlation technique was utilized to examine the interrelations among serum factors. A *P*-value less than 0.05 was deemed to hold statistical significance.


**Informed consent:** Written informed consent was provided by all patients prior to the study start.
**Ethics approval:** The present study was approved by the Ethics Committee of Hongqi Hospital Affiliated to Mudanjiang Medical College (No. 201602MD15). All procedures were performed in accordance with the ethical standards of the Institutional Review Board and The Declaration of Helsinki, and its later amendments or comparable ethical standards.

## 3 Results

### 3.1 Clinical features

Table 1 records baseline characteristics of all subjects, indicating no significant differences in age or gender among IS patients compared with healthy control subjects. In addition, ischemic lesion volume was significantly larger and NIHSS score was higher in IS patients than in healthy controls, suggesting that there was comparability between healthy controls and IS patients, and that IS patients had significant lesions and neurological impairment.

**Table 1:** Baseline characteristics of ischemic stroke patients and healthy control subjects

Characteristics	IS (*n* = 86)	HC (*n* = 32)	*P* value
Male sex no. (%)	51 (59.3%)	19 (59.3%)	0.854
Age (year)	66 (62, 68)	66 (63, 69）	0.586
Comorbidity			
Hypertension	71 (83%)	26 (81.1%)	0.235
Atherosclerosis	76 (88.3%)	9 (28.1%)	0.0502
Diabetes mellitus	65 (82%)	24 (75.0%)	0.412
Ischemic lesion volume (cm^3^)	9.36 ± 1.17	0.12 ± 0.04	0.012
NIHSS scores	8.56 ± 2.36	1.25 ± 3.14	0.0247

Data are expressed as mean ± standard deviation or *N* (%). *P* < 0.05.

### 3.2 Abnormality of serum miR-142-5p, NSE, and S100β in serum of IS patients

**Figure 1:**
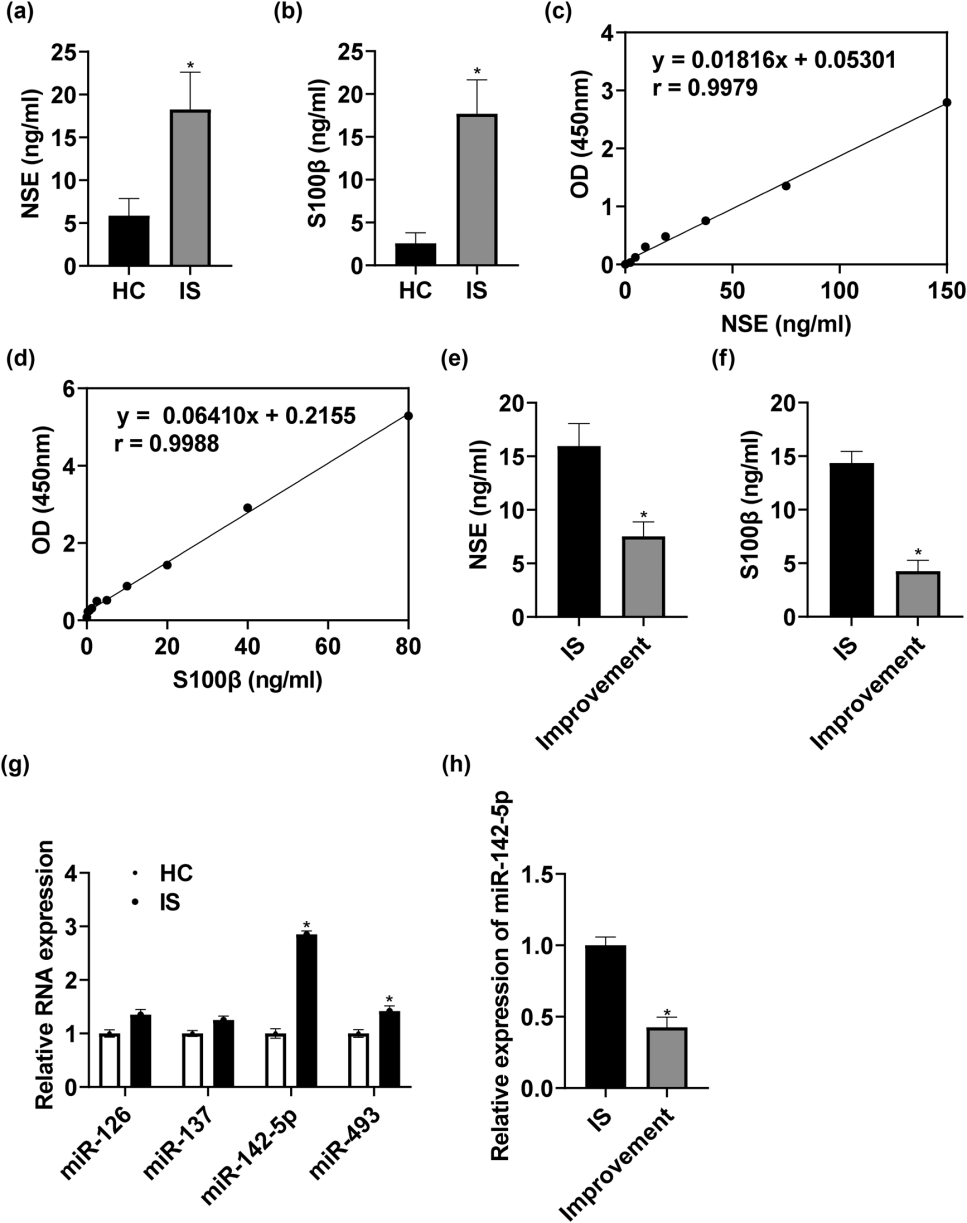
Serum levels of NSE, S100β, and four miRNAs in IS patients before and after treatment by ELISA and RT-qPCR. (a, b) ELISA to detect the concentrations of NSE and S100β in the serum of HC and IS patients; (c, d) standard curves of NSE and S100β; (e, f) ELISA to detect the concentrations of NSE and S100β in the serum of patients before and after the improvement; (g) qRT-PCR to detect the expression levels of the four miRNAs in the HC and IS patients; (h) qRT-PCR to detect the expression levels of the four miRNAs in the IS H: qRT-PCR to detect the expression levels of four miRNAs in patients with IS before and after improvement.

To screen for specific biomarkers in the serum of IS patients, we collected and analyzed biomarkers associated with nerve damage and miRNA associated with inflammation in IS patients before admission and after treatment. Results showed that before admission, serum NSE (18.25 ng/mL) and S100β (17.69 ng/mL) were higher in IS patients than in healthy volunteers (Figure 1a and b, *P* < 0.05). After 2 weeks of admission, 21 patients with no obvious changes in symptoms and signs were divided into the control group, and 65 patients with obvious improvement in their conditions and signs, who were able to move around with a cane or basically take care of themselves were divided into the improvement group. The OD values of NSE and S100β were measured by ELISA, and the standard curves were plotted to calculate the levels of NSE and S100β (Figure 1c and d). The results showed a decrease in both neuromarkers, with a reduction of approximately 53% in NSE (Figure 1e, *P* < 0.05; 95% CI [4.85, 10.19]) and approximately 70% in S100β (Figure 1f, *P* < 0.05; 95% CI [2.28, 6.24]) in the improvement group compared to the control group.

**Figure 2:**
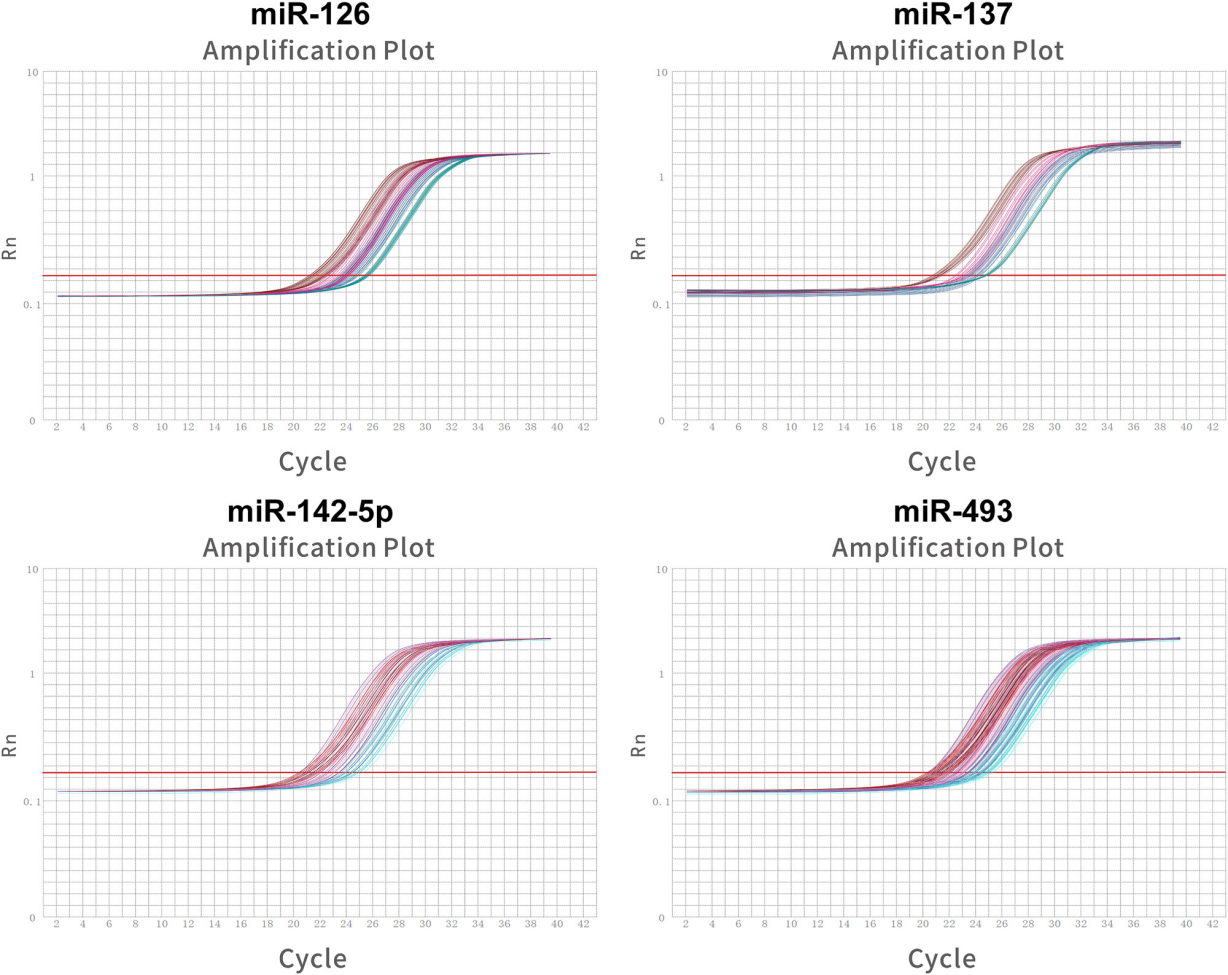
Amplification curves were generated based on the fluorescence signal and cycle number of miRNAs.

In addition, four miRNAs of interest in the serum of IS patients were analyzed. Amplification curves were constructed based on fluorescence signal and the number of cycles (Figure 2). RT-qPCR determined that the difference between serum miR-126 and miR-137 was not statistically significant, miR-142-5p and miR-493 was abnormally increased in IS patients. Serum miR-493 levels in IS patients were 1.42 times those of healthy controls (*P* < 0.05; 95% CI [1.23, 1.61]), miR-142-5p level was most significantly increased, which was 2.85 times those of healthy controls (Figure 1g, *P* < 0.05; 95% CI [2.72, 2.98]). Subsequently, miR-142-5p levels in patients after 2 weeks of admission were assessed. There was a 57.5% reduction in miR-142-5p compared with control patients (Figure 1h, *P* < 0.05; 95% CI [0.285, 0.565]). In summary, miR-142-5p, NSE, and S100β in serum of IS patients were abnormally elevated, and their levels decreased with the improvement of the disease.

### 3.3 miR-142-5p in serum of IS patients is positively correlated with NSE, S100β, and NIHSS

**Figure 3:**
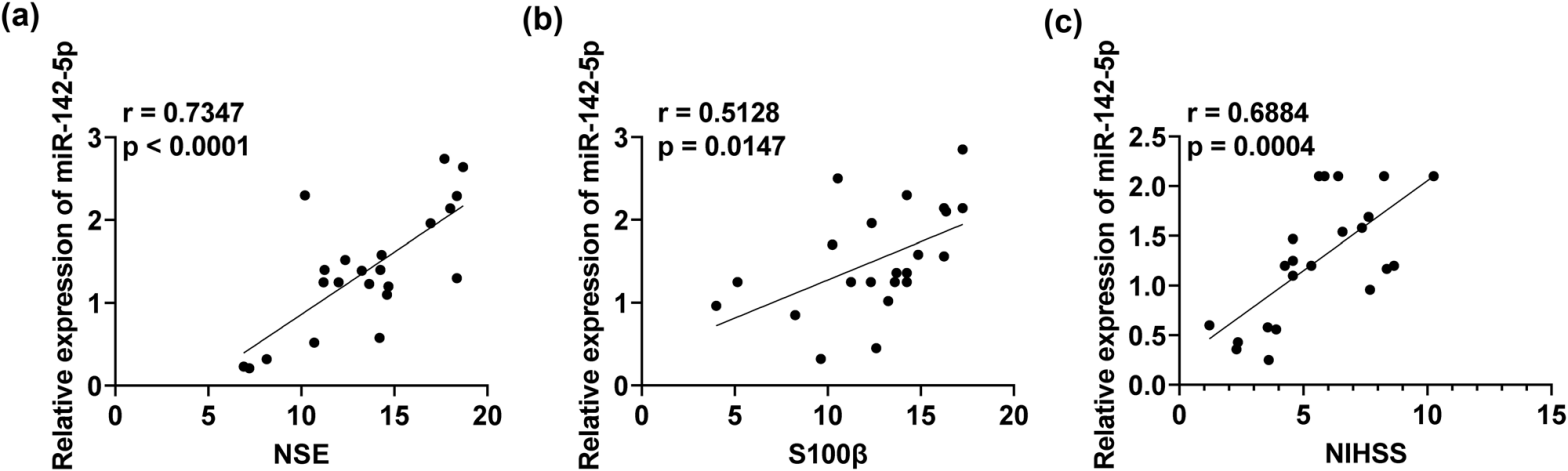
Correlation analysis of miR-142-5p with NSE, S100β, and NIHSS in serum of IS patients. (a, b) Correlation analysis of miR-142-5p with NSE and S100β in serum of IS patients; (c) Correlation analysis of miR-142-5p with NIHSS in serum of IS patients.

miR-142-5p was positively correlated with NSE (rs = 0.7347, *P* < 0.001) and S100β (rs = 0.5128, *P* = 0.0147) in IS patients (Figure 3a and b), as well as with NIHSS scores (rs = 0.6884, *P* = 0004) (Figure 3c), indicating that miR-142-5p could reflect nerve damage in IS patients to a certain extent.

## 4 Discussion

IS may result in permanent disability or death through induction of neuronal damage. However, the current diagnosis and treatment of IS more traditionally limited. Potential candidates for biomarkers of brain tissue injury, such as NSE and S100β, and it may be possible to detect the extent of cerebral infarction by measuring these serum markers [20]. Meanwhile, circulating miRNAs can be non-invasive diagnostic and prognostic biomarkers for many diseases including IS. Therefore, this study analyzed the association between potential circulating RNA and the neurobiochemical markers NSE and S100β. A significant correlation was found between serum miR-142-5p levels and NSE and S100β in patients with IS, suggesting that miR-142-5p may be a biomarker of neurological injury.

miRNAs are considerable modifiers in a variety of biological processes, including cell proliferative and apoptotic activities. Nerve injury mainly involves complex cell events related to apoptosis, inflammation, and oxidative stress pathways. Several studies have shown that miR-142-3p responds to different pathological stimuli, such as neuropathic pain and simian immunodeficiency virus encephalitis, by regulating the expression of pro-inflammatory mediators [21,22]. In a study of cerebral ischemia/reperfusion (I/R) injury, miR-142-5p is shown to be induced in hippocampal neurons by oxygen–glucose deprivation and reoxygenation treatment, and is also involved in Alzheimer’s disease pathology [23,24]. In this study, we determined miR-493 and miR-142-5p, whose expression levels were significantly dysregulated in the serum of IS patients, and miR-142-5p was selected for this study because of its significantly higher up-regulation level than miR-493. miR-142-5p was abnormally elevated after IS onset, and was inhibited after disease improvement. It suggests that aberrant expression of miR-142-5p promotes nerve damage and is a key regulator. This observation is consistent with a previous study, which demonstrates through *in vivo* experiments that downregulation of miR-142-5p may play a neuroprotective role against isoflurane-induced neurological impairment [25]. This directly specifies the important role of miR-142-5p in neurological impairment, which is involved in the process of nerve damage. In summary, this study suggests that miR-142-5p may be a novel miRNA involved in IS development.

NSE, a dimeric isoenzyme of the glycolytic enzyme enolase, is found in the cytoplasm of neurons. When the plasma membrane is functionally or structurally compromised, NSE is released from damaged neurons [26,27]. Kurakina et al. found a positive correlation between NSE levels and ischemic lesion volume and severity of neurological symptoms in patients 48 h after stroke onset [28] to determine its high predictive value in determining severity of adverse neurological events and early neurobehavioral outcomes. S100β is a substance unique to the brain. Large volumes of S100β are released into the blood and cerebrospinal fluid by astrocytes after brain tissue is injured, indicating that this protein is essential for nerve regeneration. There is a correlation between serum S100β protein level and infarct volume on Days 1–4 of ischemia [29,30], which can predict the severity of brain injury and survival and provide value for neurologic examination and neuroradiologic findings. S100β levels in the cerebrospinal fluid of patients with IS are also positively correlated with stroke severity [31]. This study also confirmed that NSE and S100β were upregulated in serum of IS patients, and were decreased in the patients after treatment. Consistent with previous studies, the expression levels of NSE and S100β reflect neurological damage in patients with IS. Meanwhile, Pearson analysis determined that miR-142-5p was positively correlated with NSE, S100β, and NIHSS, indicating that NSE, S100β levels and NIHSS scores were significantly higher with elevated miR-142-5p expression.

However, some limitations existed in this study. The sample size included in this study was small, and the results may be biased. The exact conclusions still need to be further confirmed in later studies with multicenter, long follow-up, and large samples Only four miRNAs were analyzed in IS serum. Therefore, future work needs to detect more possible miRNAs. Meanwhile, further research is needed on miR-142-5p’s potential as a biomarker of IS. In addition, only a preliminary discussion has been conducted in this article. Regarding the role of miR-142-5p on the regulation of downstream signaling pathway molecules and its effect on the levels of NSE and S100β are involved, more in-depth studies will be carried out subsequently to further elucidate the specific mechanisms of IS biomarkers and to provide the basis for clinical applications.

## 5 Conclusion

This study confirmed for the first time that miR-142-5p is abnormally upregulated in the serum of IS patients, and that miR-142-5p is positively correlated with the serum neurorelated markers NSE and S100β, suggesting that miR-142-5p is a biomarker of nerve injury and is involved in the development of IS.
